# Effect of two artificial aging protocols on color and gloss of single-shade versus multi-shade resin composites

**DOI:** 10.1186/s12903-022-02351-7

**Published:** 2022-08-01

**Authors:** Aiah A. El-Rashidy, Rasha M. Abdelraouf, Nour A. Habib

**Affiliations:** Biomaterials Department, Faculty of Dentistry, Cairo University, Cairo, 11562 Egypt

**Keywords:** Single-shade resin composite, Artificial aging, Immersion, Thermocycling, Color, Gloss

## Abstract

The long-term color stability and gloss retention of resin composites are among the crucial factors that affect the clinical longevity of esthetic restorations, especially in anterior teeth. This study evaluated the effect of artificial aging by immersion in different storage media and thermocycling on color and gloss of dental single-shade resin composite (Omnichroma) versus multi-shade one (Filtek Z350XT). One hundred resin-composite disc-shaped specimens were used, 50 from each group, Omnichroma and Filtek Z350XT. Ten specimens from each material acted as control group (incubated in saliva). For each material, 40 specimens were divided according to the artificial-aging protocol (immersion at 37 °C for 12 days or thermocycling for 10,000 cycles) and storage media (tea, red wine). Color and gloss were measured before and after artificial aging. Color difference (∆E_00_) was compared with perceptibility threshold and acceptability threshold. Data were statistically analyzed; independent t test was used to compare results between two tested materials, while two-way ANOVA was used to compare results among the different immersion media within the same material. Artificial aging (immersion or thermocycling) in tea and red wine led to significant color changes and gloss reduction in both materials (*P* < 0.05), in contrast to control group. Red wine produced highest color differences. Both dental resin-composites; the single-shade (Omnichroma) and multi-shade (Filtek Z350XT) displayed unacceptable discoloration and gloss reduction after artificial-aging in tea and red-wine by immersion or thermocycling simulating one-year clinical-service.

## Introduction

Resin-based dental composite materials are widely used in dentistry as a direct aesthetic tooth-colored restorative material [1]. Due to the increased esthetic demands, clinicians are faced with the challenge to reproduce the color of natural teeth [2]. The shade of the available resin composites is generally described based on the Munsell color system, which classified colored objects by three color dimensions: hue, chroma and value [3]. Most manufacturers of resin composites label the shade of their products following the VITA classical shade system (VITA Zahnfabrik, Sackingen, Germany). These multi-shade resin composites have letters, e.g. A, B, C, and D represents the hue, while numbers, e.g. 1, 2, 3, and 4 denote chroma and value [4]. Unfortunately, the presence of such variable shades may complicate the shade matching procedure and increases the cost and chairside time [5]. Single-shade (universal shade) resin composite has been introduced to replace various shades by providing shade matching for all tooth colors using only one shade with chameleon or blending effect [5–7].

According to Ismail and Paravina [8], the scientific term color adjustment describes the ability of resin composite materials to adjust their color to the color of surrounding enamel and dentin, similarly the terms color blending/shifting/assimilation, where all are considered more scientific terms than the dental jargon “chameleon effect” [8]. Recently, a single-shade composite, Omnichroma, was developed by Tokuyama Dental, which is supposed to match all Vita classical shades, from A1 to D4. Omnichroma was shown by Sanchez et al. [9] to exhibit the highest instrumental and visual color adjustment potential for each of the 16 VITA classical A1-D4 shades, compared with Filtek Z350 XT, TPH Spectra, Herculite Ultra, and Tetric EvoCeram. This indicates the potential of Omnichroma to blend with the surrounding enamel and dentin, thus improving the esthetic appearance and simplify the shade matching procedure [9]. However, in a study be Iyer et al. [6], Omnichroma displayed inferior visual and instrumental shade matching ability to bi-layered acrylic teeth with A2, B1, B2, C2, and D3 shades, as compared to a multi-shade composite material, which could limit their use in highly esthetic areas [6].

The long-term color stability and gloss retention of resin composites are among the crucial factors that affect the clinical longevity of esthetic restorations, especially in anterior teeth [10, 11]. A major disadvantage of resin composite restorations is its susceptibility to staining, discoloration and loss of surface gloss caused by the aging process in the oral environment. Color instability is considered among the main reasons for restorations replacement, especially in anterior teeth [10]. Color changes of composite resin materials are affected by extrinsic and/or intrinsic factors [12]. Intrinsic factors affecting color stability is mainly related to the materials̕ composition [13]. On the other hand, extrinsic factors include the absorption of colorants contained in foods and beverages such as tea, coffee, red wine, as well as smoking or poor oral hygiene [10]. Yet, an interaction exists between the intrinsic and extrinsic factors. For example, the resin composites̕ composition (resin matrix structure and filler characteristics) affects the susceptibility to extrinsic staining [13].

As mentioned earlier, maintenance of surface gloss is among the major esthetic factors that affect the clinical longevity of the restoration. Reduction in surface gloss is often related to wear or material deterioration due to aging process [14]. Several in vitro methods are used to simulate aging in the oral environment, including immersion in different staining solutions and thermocycling.

Based on our knowledge, little information is available about the effect of staining and aging on the color stability of Omnichroma single-shade composite. Thus, the aim of this in vitro study is to evaluate and compare the effect of aging of two commercial resin composites, one single-shade resin composite (Omnichroma) versus a multi-shade one (Filtek Z350 XT) in different staining solutions (artificial saliva, tea, and red wine) with/without thermocycling, on their color stability and gloss retention. The null hypothesis is that storage in staining agents will not affect the color and gloss of the esthetic restorative materials and no difference between results obtained after immersion and that after thermocycling.

## Materials and methods

### Materials

Two commercially available resin composite materials were used (Table 1).

**Table 1:** Materials used in this study

Product	Manufacturer	Filler type	Filler content		Matrix composition	Shade	Lot
			wt%	vol%			
Omnichroma	Tokuyama Dental, Tokyo, Japan	Uniform sized supra-nano spherical filler (260 nm spherical SiO_2_-ZrO_2_) and composite filler	79%	68%	UDMA*, TEGDMA**	Universal	016E21
Filtek Z350 XT	3 M ESPE, Minnesota, USA	non-agglomerated/non-aggregated 20 nm silica filler, non-agglomerated/non-aggregated 4 to 11 nm zirconia filler, and aggregated zirconia/silica cluster filler (comprised of 20 nm silica and 4 to 11 nm zirconia particles)	78.5%	63.3%	Bis-GMA***, UDMA, TEGDMA, Bis-EMA(6)† PEGDMA††	A2B	NC93014

*UDMA urethane dimethacrylate, **TEGDMA triethylene glycol dimethacrylate,***BisGMA bisphenol A diglycidildimethacrylate, †*Bis-EMA* Ethoxylatedbisphenol A dimethacrylate. ††*PEGDMA* polyethylene glycol dimethacrylate

### Methods

#### Specimen preparation

A total of 100 resin composite disc-shaped specimens was used in this study (diameter = 8 mm and thickness = 1 mm). Fifty discs from each resin material were prepared using a Teflon mold following the manufacturer's instructions and were cured using a light-emitting diode (LED) curing unit [Mini LED, Satelec, Acteon, France] operating with a wavelength of 400–500 nm and an intensity of 1,000 mW/cm^2^. The light intensity of the curing unit was checked frequently with a spectroradiometer (Demetron Research Corp. USA). Each specimen was exposed to 20 s curing cycles for the top surface as well as bottom surface. A clear plastic strip was placed over the Teflon mold to prevent the formation of resin oxygen rich layer after curing. A glass slide (1 mm thick) was placed over the plastic strip to allow standardization of the specimens̕ thickness and the distance from the specimens̕ surface to the curing light-tip.

#### Specimens grouping

The 50 specimens from each resin composite material were randomly divided into five groups, ten each, according to the used staining solution and aging protocol (Table 2):

- *Group 1:* specimens immersed in artificial saliva at 37 °C for 12 days (control),
- *Group 2:* specimens immersed in tea at 37 °C for 12 days; tea was prepared as follows: one tea bag immersed for 5 min. in 200 ml boiling water (Lipton Yellow Label; Unilever; UK),
- *Group 3:* specimens immersed in red wine (Omar El Khayam; Egypt) at 37 °C for 12 days.
- *Group 4:* specimens subjected to thermocycling in tea (prepared as previous). The specimens underwent thermocycling for 10,000 cycles over a dwell time of 60 s and a transfer time of 10s [15], between 37 °C and 57 °C.
- *Group* 5: specimens subjected to thermocycling in red wine. The specimens underwent thermocycling for 10,000 cycles over a dwell time of 60 s and a transfer time of 10s [15] between 12 °C and 37 °C.

**Table 2:** Specimens grouping

	Staining solution	Condition	Temperature	Duration
Gp1	Saliva	Constant temperature immersion	37 °C	12 days
Gp2	Tea	Constant temperature immersion	37 °C	12 days
Gp3	Red wine	Constant temperature immersion	37 °C	12 days
Gp4	Tea	Thermocycling	37–57 °C	10,000 cycles
Gp5	Red wine	Thermocycling	12–37 °C	10,000 cycles

#### Immersion and thermocycling processes

For the constant temperature immersion groups (Gp1, 2 & 3), each specimen was stored in a separate vial, containing 3 ml of the staining solution. All specimens were stored in the respective staining solution in an incubator at 37 °C (BTC, Egypt) and specimens were rinsed with distilled water and immersed in fresh solutions every 3 days to avoid any bacterial or fungal contamination [16, 17]. Specimens were stored for 12 days in each staining solution. At the end of the immersion period, specimens were rinsed with distilled water, wiped with gauze and air-dried.

For the two thermocycling groups (Gp 4 & 5), the tea and red wine were used as staining solutions in temperatures simulating the actual clinical condition The specimens underwent thermocycling between 37 °C and 57 °C for the tea group and between 12 °C and 37 °C for the red wine group. For each group, the number of cycles was 10,000 cycles over a dwell time of 60 s and a transfer time of 10 s.

#### Color difference determination

For each specimen, a baseline color (T0) was measured before aging using a spectrophotometer (Cary 5000, Agilent Technologies, USA). Then after aging, the final (Tf) color measurements were made for each specimen after immersion or thermocycling.

The color difference (ΔE_00_) was measured according to the CIEDE2000. This is the newest color difference formula intended to correct the differences between the measurement result and visual evaluation, which was the weak point in the L*a*b* color space [18]. The calculation is based on the following equation:

$$\Delta {\text{E}}_{00} = \sqrt {\left( {\frac{{\Delta {\text{L}}^{\prime } }}{{{\text{k}}_{{\text{L}}} + {\text{S}}_{{\text{L}}} }}} \right)^{2} + \left( {\frac{{\Delta {\text{C}}^{\prime } }}{{{\text{k}}_{{\text{C}}} + {\text{S}}_{{\text{C}}} }}} \right)^{2} + \left( {\frac{{\Delta {\text{H}}^{\prime } }}{{{\text{k}}_{{\text{H}}} + {\text{S}}_{{\text{H}}} }}} \right) + \left( {{\text{R}}_{{\text{T}}} \left( {\frac{{\Delta {\text{C}}^{\prime } }}{{{\text{k}}_{{\text{C}}} + {\text{S}}_{{\text{C}}} }}} \right)\left( {\frac{{\Delta {\text{H}}^{\prime } }}{{{\text{k}}_{{\text{H}}} + {\text{S}}_{{\text{H}}} }}} \right)} \right)}$$

where ∆L: lightness difference, ∆C: saturation difference, ∆H: hue difference, with correction using weighing coefficients (SL, SC, and SH) and constants called parametric coefficients (kL, kC, & kH).

Measurements were carried out at wavelengths ranging from 380 to 780 nm at 1 nm intervals. A 2° observer function was used with CIE illuminant D65. Measurements were carried out against a black background. The calculation of ΔE_00_ was done by using the Excel spreadsheet implementation of the CIEDE2000 color difference formula provided by Sharma [18].Color difference (ΔE_00_) was correlated to perceptibility and acceptability thresholds. Perceptibility threshold (PT) refers to the smallest color difference that could be noticed by an observer (when ΔE_00_=0.8). While, acceptability threshold (AT) refers to the difference in color that observers could consider unacceptable, requiring color correction (when ΔE_00_ =1.8). These thresholds had been determined previously when 50% of observers perceived the color difference (PT) or considered it unacceptable (AT) [19].

#### Gloss change determination

Baseline (G0) and final (Gf) gloss measurements were made for each specimen before and after aging using glossmeter (ZGM 1130, Zehntner GmbH Testing Instruments, Switzerland). The position of each specimen was maintained using a black opaque plastic mold placed over the specimens during the measurements to eliminate the influence of the ambient light and maintain the position of the specimen during repeated measurements. Five measurements per specimen were performed at 60° light incidence and reflection angles. The five readings were averaged to obtain a single value for each specimen.

#### Sample size calculation

A study of a continuous response variable from independent control and experimental subjects with 1 control(s) per experimental subject was planned. In a previous study by Poggio et al. in 2017 [10], the response within each subject group was normally distributed with standard deviation 8.5. If the true difference in the experimental and control means is 11, we will need to study 10 experimental subjects per each group and 10 control subjects to be able to reject the null hypothesis that the population means of the experimental and control groups are equal with probability (power) 0.8. The Type I error probability associated with this test of this null hypothesis is 0.05. Sample size was calculated using PS (Power and Sample size program), version 3.1.2 for windows.

#### Statistical analysis

Data was analyzed using SPSS, version 26 for windows (SPSS Inc., Chicago, IL). Data showed normal distribution using Kolmogrov Smirnov test and Shapiro Wilk test. Continuous data were described using mean and standard deviation. Intergroup comparison was performed using independent t test, while intragroup comparison was performed using two-way ANOVA followed by Tukey post-hoc test and paired t test when appropriate. *P* value less than or equal to 0.05 was considered statistically significant with a confidence level of 95% and a power of 80%, and all tests were two tailed.

## Results

### Color measurement

**Fig. 1:**
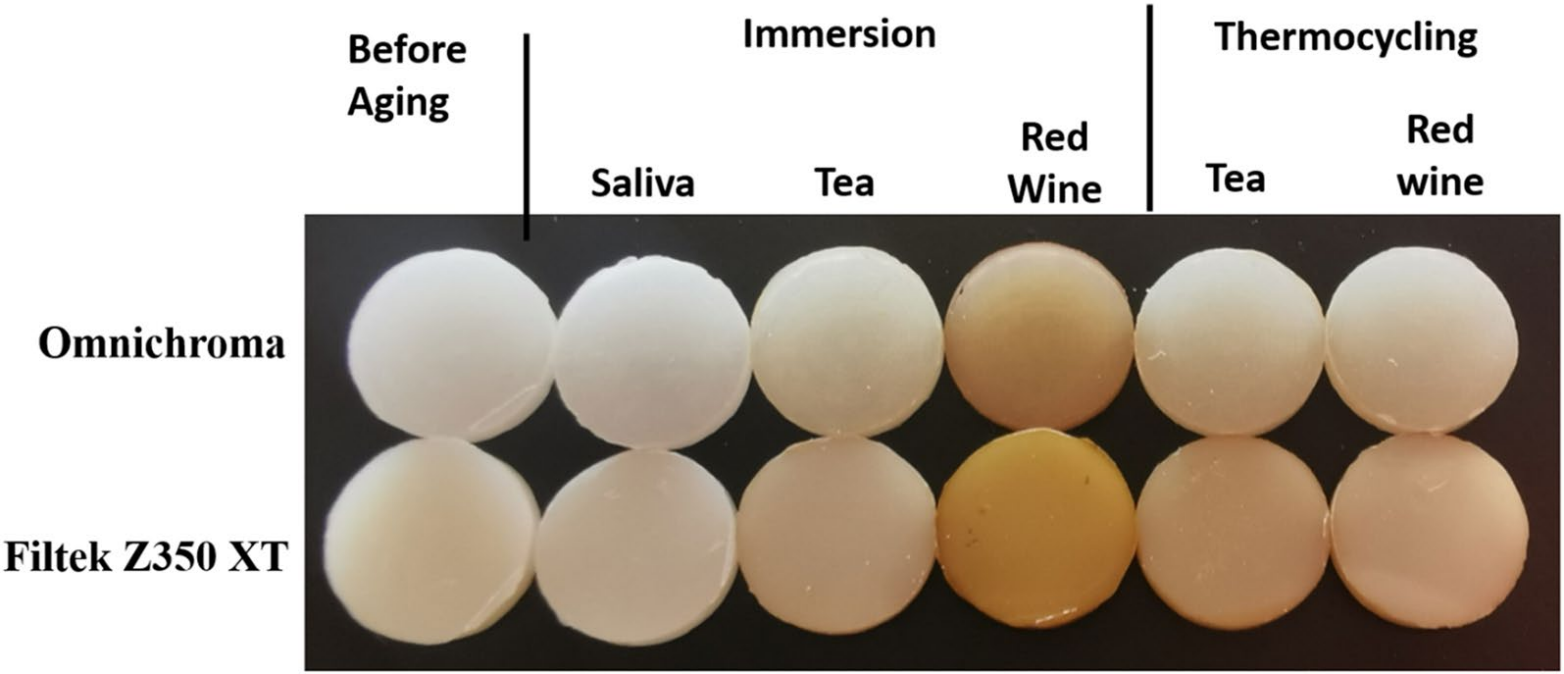
Photograph of representative specimens from each group

Figure 1 showed representative specimens from the single-shade (Omnichroma) and the multi-shade (Filtek Z350 XT) before and after artificial aging; immersion in saliva (control), tea and red wine, as well as thermocycling in tea and red wine.

### Effect of immersion on color difference (∆E_00_)

Intergroup comparison between both composites have shown no statistically significant difference in saliva (*P* = 0.6102) and showed statistically significant difference in tea and red wine (*P* < 0.0001), (Table 3). The color changes in the single shade (Omnichroma) was significantly higher than the multi-shade (Filtek Z350 XT).

**Table 3:** Mean and standard deviation of ∆E_00_ after immersion of both materials within each storage medium

Intervention	Omnichroma		Filtek Z350 XT		*P* value
Storage medium	Mean	SD	Mean	SD	
Saliva	0.86^a^	0.07	0.88^a^	0.08	*P* = 0.6102
Tea	4.5^b^	0.2	3.64^b^	0.27	*P* < 0.0001*
Red wine	8.12^c^	0.29	6.52^c^	0.22	*P* < 0.0001*
*P* value	< 0.001*		< 0.001*		

Means with different letters in the same column indicate statistically significance difference while means in the same row has no letters as they are two groups only. *Corresponds to statistically significant difference

Intragroup comparison within Omnichroma or Filtek Z350 XT have shown statistically significant difference between different storage media (*P* < 0.001), where the red wine caused the highest color changes, followed by tea and the least was saliva, Table 3.

In both materials, the color difference after immersion in saliva was just above perceptibility threshold, while immersion in tea and red wine led to unacceptable color difference.

### Effect of thermocycling on color difference (∆E_00_)

Intergroup comparison between both composites have shown no statistically significant difference in tea and red wine (*P* = 0.0982 and *P* = 0.3635) respectively (Table 4). Intragroup comparison within Omnichroma or Filtek Z350 XT have shown statistically significant difference in the different storage media (*P* < 0.001), where the red wine caused significantly higher color changes than tea (Table 4). Both tea and red wine led to unacceptable color difference after thermocycling.

**Table 4:** Mean and standard deviation of ∆E_00_ after thermocycling of both materials within each storage medium

Intervention	Omnichroma		Filtek Z350 XT		*P* value
Storage medium	Mean	SD	Mean	SD	
Tea	3.24^a^	0.2	3.39^a^	0.19	*P* = 0.0982
Red wine	4.3^b^	0.26	4.4^b^	0.21	*P* = 0.3635
*P* value	*P* < 0.001‏‏*		*P* < 0.001*		

Means with different letters in the same column indicate statistically significance difference while means in the same row has no letters as they are two groups only. *Corresponds to statistically significant difference

### Effect of aging method on ∆E_00_

There were significant differences between color changes (∆E_00_) resulted after immersion and that caused by thermocycling either in Omnichroma or Filtek Z350 XT specimens ((*P* < 0.05) (Table 5). Generally, aging by immersion resulted in significantly higher color changes compared to the thermocycling.

**Table 5:** Mean and standard deviation of ∆E_00_ after aging of both materials within each storage medium:

Intervention	Omnichroma				*P* value	Filtek Z350 XT				*P* value
	After immersion		After thermocycling			After immersion		After thermocycling		
Storage medium	Mean	SD	Mean	SD		Mean	SD	Mean	SD	
Tea	4.5	0.2	3.24	0.2	*P* < 0.0001*	3.64	0.26	3.39	0.19	*P* = 0.0230*
Red wine	8.12	0.28	4.3	0.26	*P* < 0.0001*	6.52	0.22	4.4	0.21	*P* < 0.0001*

^*^Corresponds to statistically significant difference

### Changes in CIE Lab parameters

**Fig. 2:**
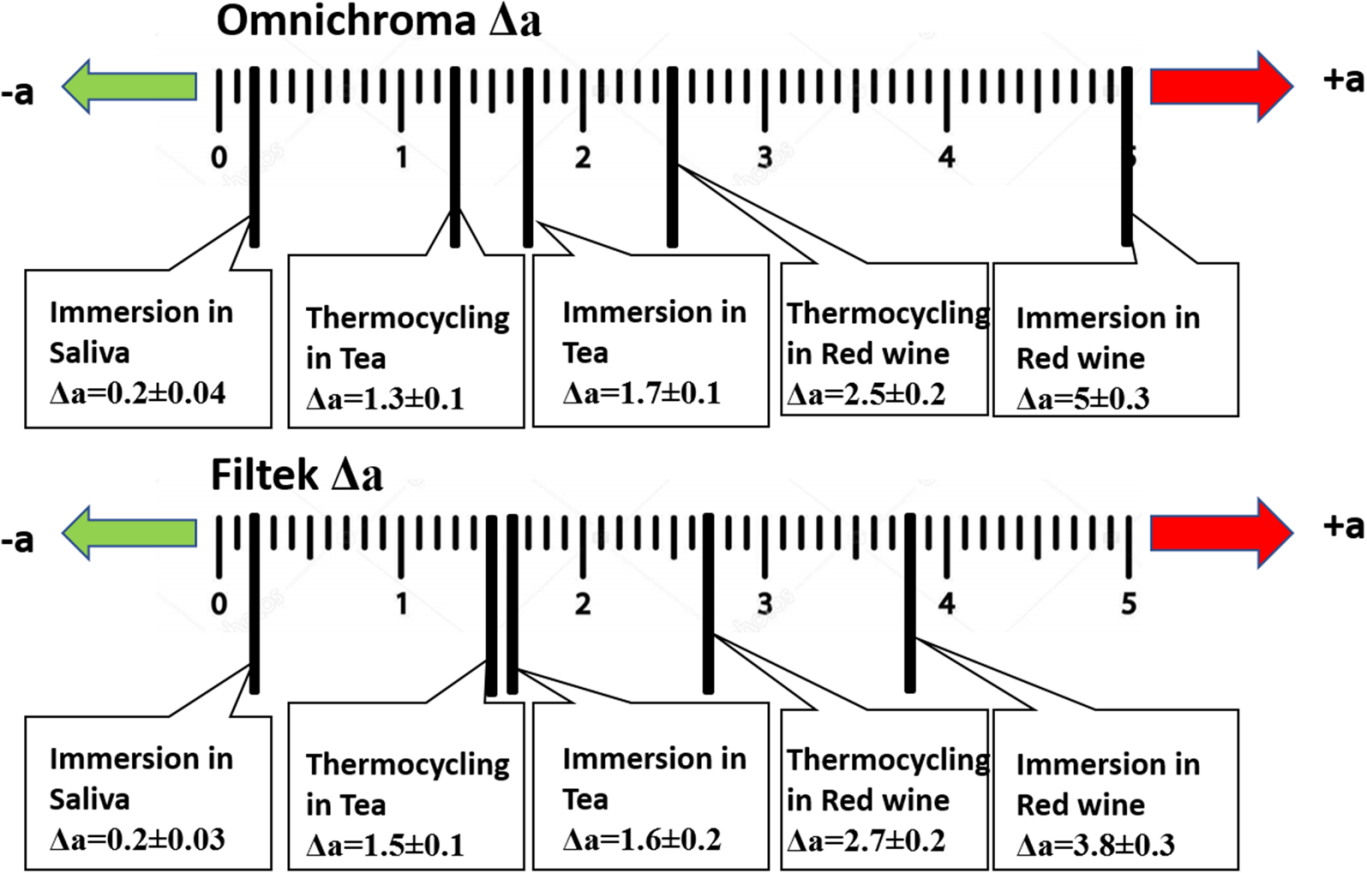
Changes in hue across the red-green axis (Δa)

Changes in hue across the red-green axis (Δa) (Fig. 2), showed that the two aging techniques led to a more reddish color in all storage media in both materials.

**Fig. 3:**
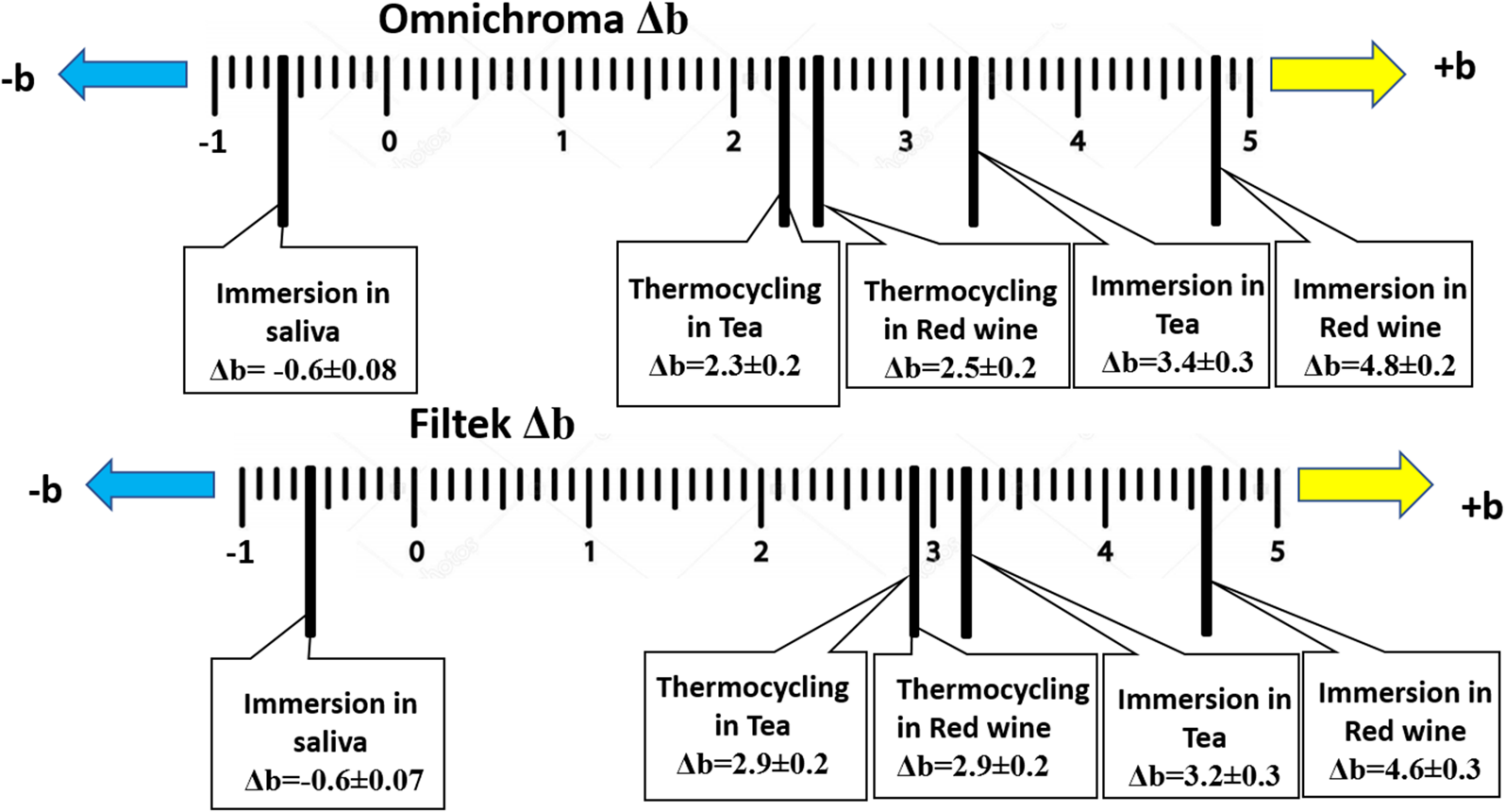
Changes in hue across the yellow-blue axis (Δb)

Changes in hue across the yellow-blue axis (Δb) (Fig. 3), showed that the two aging techniques led to a more yellowish color after storage in tea and red wine in both materials. Meanwhile, immersion in saliva caused a negative b value (Δb =  − 0.6) indicating a slight reduction in yellow content.

**Fig. 4:**
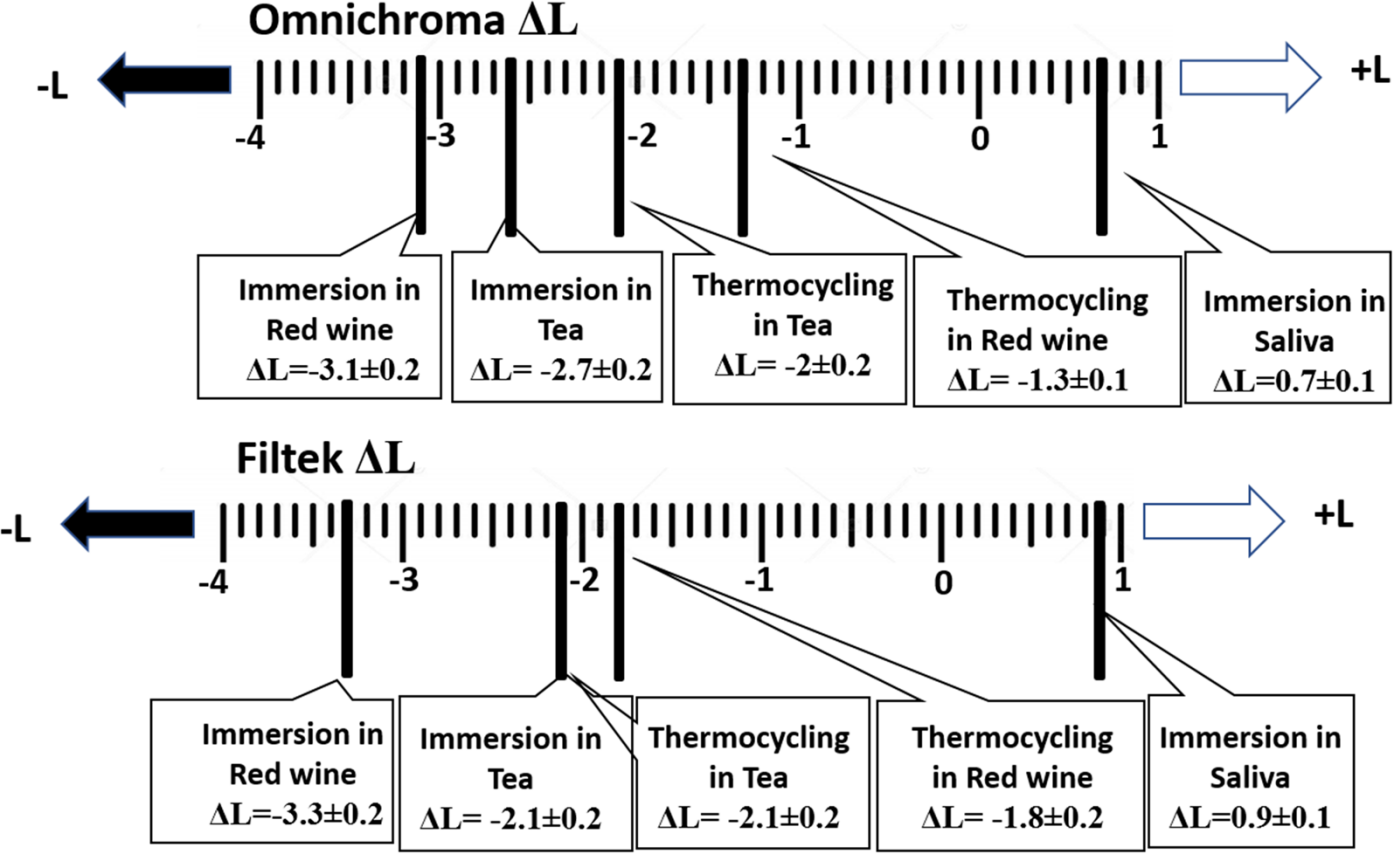
Changes in lightness (ΔL)

Changes in lightness (Δ L) is shown in Fig. 4. There was a reduction in value (negative ΔL) after aging in tea and red wine in both materials. This indicates that the materials become darker. Contrary, immersion in saliva gave positive ΔL values (ΔL = 0.7 in Ominchroma and 0.9 in Filtek) indicating that the specimens became lighter.

### Gloss measurement

#### Effect of immersion on gloss

Before aging, the gloss of Filtek Z350 XT (37.8 ± 2.9) was significantly higher than Omnichroma (31.96 ± 2.38), *P* < 0.0001.

The gloss of either Omnichroma or Filtek Z350 XT specimens showed significant difference before and after immersion in all storage media (*P* < 0.05) (Table 6). The gloss became significantly higher after immersion in saliva than before immersion, contrary to the immersion in tea and red wine, the gloss decreased significantly.

**Table 6:** Mean and standard deviation of gloss of both materials with each storage medium before and after immersion

Intervention	Omnichroma				*P* value	Filtek Z350 XT				*P* value
	Before immersion		After immersion			Before immersion		After immersion		
Storage medium	Mean	SD	Mean	SD		Mean	SD	Mean	SD	
Saliva	31.96^a^	2.26	32.36^a^	2.26	*P* < 0.0001*	37.64^a^	1.4	38.29^a^	1.39	*P* < 0.0001*
Tea	31.21^a^	2.76	28.23^b^	2.82	*P* < 0.0001*	37.61^a^	3.29	33.82^b^	3.1	*P* < 0.0001*
Red wine	31.74^a^	1.66	28.25^b^	1.68	*P* < 0.0001*	36.5^a^	2.72	32.23^b^	2.7	*P* = 0.0035*
*P* value	*P* = 0.752		*P* < 0.001*			*P* = 0.541		*P* < 0.001*		

Means with different letters in the same column indicate statistically significance difference while means in the same row has no letters as they are two groups only. *Corresponds to statistically significant difference

In each material, there was no significant difference between the specimens̕ gloss before immersion in the different storage media. Yet, the gloss of the specimens immersed in tea and red wine was significantly lower than those stored in saliva. No significant difference between the reduced gloss of the specimens stored in tea and that stored in red wine.

#### Effect of thermocycling on gloss

The gloss of either Omnichroma or Filtek Z350 XT specimens showed significant difference before and after thermocycling in the different storage media (*P* < 0.0001*), Table 7. The gloss became significantly lower after immersion in tea and red wine than before immersion.

**Table 7:** Mean and standard deviation of gloss of both materials with each storage medium before and after thermocycling

Intervention	Omnichroma				*P* value	Filtek Z350 XT				*P* value
	After immersion		After thermocycling			After immersion		After thermocycling		
Storage medium	Mean	SD	Mean	SD		Mean	SD	Mean	SD	
Tea	28.23	2.8	30.69	2.08	*P* = 0.0401*	33.8	3.1	35.02	3.8	*P* = 0.4482
Red wine	28.25	1.7	29.74	2.96	*P* = 0.1840	32.2	2.7	37.2	2.9	*P* = 0.0008*

Means with different letters in the same column indicate statistically significance difference while means in the same row has no letters as they are two groups only. *Corresponds to statistically significant difference

In each material, there was no significant difference between the gloss of the specimens before thermocycling in the different storage media. In addition, there was no significant difference between the specimens̕ gloss after thermocycling in tea and that thermo-cycled in red wine.

#### Effect of aging method on gloss

Although there was no significant difference in the gloss results of the specimens before immersion and that before thermocycling (*P* > 0.05), yet after aging there were significant differences in some groups (Table 8). Generally, aging by immersion resulted in lower gloss values compared to the thermocycling. However, the gloss was significantly lower after immersion in tea in the Omnichroma group than those thermocycled in tea (*P* = 0.0401*). And the gloss was significantly lower after immersion in the red wine in the Filtek Z350 XT group than those thermocycled in red wine (*P* = 0.0008*).

**Table 8:** Mean and standard deviation of gloss after aging of both materials within each storage medium

Intervention	Omnichroma				*P* value	Filtek Z350 XT				*P* value
	After immersion		After thermocycling			After immersion		After thermocycling		
Storage medium	Mean	SD	Mean	SD		Mean	SD	Mean	SD	
Tea	28.23	2.8	30.69	2.08	*P* = 0.0401*	33.8	3.1	35.02	3.8	*P* = 0.4482
Red wine	28.25	1.7	29.74	2.96	*P* = 0.1840	32.2	2.7	37.2	2.9	*P* = 0.0008*

^*^Corresponds to statistically significant difference

## Discussion

Within the limitations of this study, Omnichroma was used as a single-shade resin composite, being one of the most clinically successful single-shade composites. It is considered one of the most widely researched single-shade material, with several research work investigating its color adjustment potential [6, 9, 20–22], and optical properties [23, 24]. The color stability of Omnichroma under accelerated aging conditions using Xenon Arc UV light sources to simulate exposure to natural sunlight [25] and 1 week staining in tea [26] were also investigated. In addition, its instrumental color adjustment potential was shown to improve after 1 month storage in distilled water [27]. Yet, the color stability and gloss change of this material in different beverages under different aging conditions, according to our knowledge, are not widely investigated.

Filtek Z350 XT universal nanocomposite was used as a control group in the current study. Although having different filler and matrix systems, still, it is considered as one of the most investigated composite materials, especially regarding their color stability and gloss characteristics [4, 10, 28, 29, 29, 30]. In literature, comparing color stability between different commercial composites are commonly based on comparing materials with different compositions to assess the effect of such compositional variations on their performance [10, 28, 29, 31–35]. The A2B shade was selected as A2 is considered the most used shade clinically [36]. According to the manufacturer, most restorations can be made with just one of the Body shades, without the need for a layering placement technique.

Polymeric based materials are commonly used in dentistry [37–39]. Single-shade resin composites have been introduced in the dental market to facilitate the shade selection step and reduce the time of application performed in the other multi-shade resin composites [40, 41] Although the color and gloss of some esthetic restorative materials may be promising initially, unfortunately color changes and loss of gloss may occur by time [42], beverages may accelerate such changes [42].

For color stability evaluation, various artificial aging techniques have been introduced as immersion in different storage media or thermocycling [14]. In this study immersion was performed at 37ºC simulating the mouth temperature, for 12 days representing one-year clinically, as 24 h of immersion in vitro were reported to simulate one month *in vivo* [16, 43]. Three storage media (artificial saliva, tea and red wine) were used in immersion; artificial saliva as control while tea and red wine as representative of hot or cold drinks which may stain resin composite restorations [43].

One of the main aims of the current study was to compare the effect of thermocycling on the color and gloss changes, as in the clinical situation, dental restorations are subjected to such thermal fluctuations, rather than constant temperature. According to Porojan et al. [44], difference in the thermal expansion coefficients between the filler particles and resin matrix induce internal stresses in the material during continuous temperature changes. Also, according to Reddy et al. [45], thermal cycling increases water sorption in resin composites. Thus, aging by thermocycling was performed for 10,000 cycles, simulating one-year clinical service [46] and was conducted in temperatures simulating actual temperatures for such beverages. Although most of thermocycling procedures are performed at temperature range of 5–55 °C, this not relevant to the clinical situation during consuming different beverages. We aimed at selecting the temperature ranges from the ideal drinking temperature of tea and red wine, 57 °C and 12 °C, respectively, to the normal oral temperature which is typically 37 °C. In our opinion, this better mimic the clinical situation. Hence, prepared composite specimens underwent thermocycling between 37 °C and 57 °C for the tea group and between 12 °C and 37 °C for the red wine group. It should be noted that thermocycling was not performed in the artificial saliva group, as the oral cavity temperature is at about 37ºC. Thus, immersion was the only artificial aging technique used with the saliva group.

In this study, specimens were not polished but flat surfaces were obtained by pressing the resin between two glass slides and the restorative materials were polymerized against a Mylar strip [47]. According to Bashetty et al. [48], Mylar strip produced the smoothest of all the finishing and polishing systems when compared to one-step and multi-step polishing systems. This was performed to avoid any finishing and polishing variables. As it has been reported that the finishing and polishing step of resin composite restorations could cause surface cracks and scratches increasing resultant surface roughness and subsequently the staining liability [49]. Therefore, smooth surface by mylar strip was preferred rather than finishing and polishing process to avoid introducing new variables which may interfere with the results aiming to compare the two materials in a more standardized condition.

Color change was measured before and after aging according to the CIEDE2000 color difference (ΔE_00_) [18]. According to CIEDE2000, the perceptibility threshold was 0.8 representing the magnitude of color difference that could be detected visually [50]. While the acceptability threshold was 1.8 which is the color difference magnitude representing an unacceptable esthetic limit [50].

The null hypothesis was rejected, as the storage in staining agents affected the color and gloss of the esthetic restorative materials and there was difference between results obtained after immersion and that after thermocycling.

As a result of saliva immersion, the color changes were just above perceptibility threshold for both materials; Omnichroma and Filtek Z350 XT. In contrast to the major color changes produced by tea and red wine which were above the acceptability threshold. This may be attributed to their high staining effect as verified in the previous studies [10, 28, 29, 31–33, 43, 51].

Tea led to unacceptable color changes in both materials by the two aging methods (immersion and thermocycling). This color changes may be attributed to the ability of the tea molecules to penetrate deep inside the restorative materials leading to staining and material discoloration as reported in literature [31].

Red wine caused the highest discoloration among all immersion media in both resin composite materials. All color changes were high above the acceptability threshold. This may be attributed to the high concentration of pigments in red wine [52]. Tannin and anthocyanins (water soluble pigments) from grapes may promote significant color changes in the red wine groups [53, 54]. In addition, being an alcohol, this may degrade the surface of resin composite resulting in a rough surface favoring more pigment deposition and further staining [53]. This agrees with previous researches where the red wine resulted in highest discoloration in resin composites compared to other drinks [52, 53, 55].

The color changes in the multi-shade (Filtek Z350 XT) were significantly lower than the single shade (Omnichroma). This may be attributed to their resin matrix composition. The Filtek Z350 XT consisted of higher molecular weight monomers as BisEMA characterized by its low water sorption as a result of its hydrophobicity and high degree of conversion [56], in addition to Bis-GMA which increase the polymer crosslinking density [11, 57]. Contrary, Omnichroma matrix composed of lower molecular weight monomers (UDMA and TEGDMA). It has been reported that TEGDMA (hydrophilic monomer) increased water sorption hindering color stability [56].

Although the Filtek Z350 XT displayed lower discoloration than Omnichroma after aging in beverages, yet both materials suffered from color instability. The color instability in Filtek Z350 XT was reported in the literature and was attributed to the infiltration of colorants and water at the interface between the non-perfectly silanized nano-aggregated particles and resin matrix [58]. While the staining susceptibility of Omnichroma had just been discussed before.

Comparing the effect of the two artificial aging methods (immersion and thermocycling) on color changes, there were significant differences between color changes (∆E_00_) resulted after immersion in general and that caused by thermocycling in both materials. Generally, aging by immersion resulted in significantly higher color changes compared to the thermocycling. This may be attributed to the static nature of immersion technique, in contrast to the dynamic effect of the thermocycling, where the mobility of the immersion media may have a washing effect on some of the deposited stains [59]. In addition to the continuous effect of immersion in contrast to the intermittent action of thermocycling as a result of periods of rest during transfer time between the two temperatures (time interval between dwells) [60]. Thermocycling could simulate the intermitted action of sipping of drinks occurring in real life. It should be noted that both artificial aging methods led to significant color differences, thus immersion could be considered a simpler artificial aging method for detecting color changes compared to the equipped thermocycling technique.

Changes in hue across the red-green axis (Δa) and the yellow-blue axis (Δb) showed that the two aging techniques led to a more reddish and yellowish color in both materials. The immersion in red wine caused the maximum reddish/ yellowish color change, while the least was immersion in saliva. Aging in red wine led to a higher reddish/yellowish discoloration compared to tea in both materials. This may be attributed to the high pigments content in red wine as discussed previously [53, 54]. Immersion led to more reddish/yellowish color change than thermocycling either in tea or red wine. This might be attributed to pronounced effect of immersion in aging as discussed previously. It was noticed that immersion in saliva caused a negative b value (Δb =  − 0.6) indicating a slight reduction in yellow content, this may be attributed to the dilution effect due to absorption of saliva [42, 61, 62].

There was a reduction in value (negative ΔL) after aging in tea and red wine in both material by immersion and thermocycling. This indicates that the materials become darker which may be due to discoloration by pigments [31, 53, 54]. The effect of immersion in different storage media was more pronounced than thermocycling as discussed previously. It should be noted that immersion in saliva gave positive ΔL values indicating that the specimens became lighter. Again, this may be due to the nature of saliva which is devoid of pigments and its diluting effect [42, 61, 62].

In general, the gloss of dental restorations is associated with shiny smooth surfaces that reflected light in a specular manner [42]. In this research, the gloss of Filtek Z350 XT before aging was significantly higher than Omnichroma. This may be attributed to variation in their filler size [63]; the smaller nanofillers size in Filtek Z350 XT (20 nm silica and 4–11 nm zirconia) may lead to lower surface roughness and therefore better surface finish and gloss retention compared to the larger submicron fillers in Omnichroma (260 nm spherical SiO_2_-ZrO_2_).

The gloss of either Omnichroma or Filtek Z350 XT specimens showed significant difference before and after aging (immersion and thermocyclying) in the different storage media. The gloss became significantly lower after immersion or thermocycling in tea and red wine than before. It had been documented in previous studies that beverages̕ acidity may affect the gloss of restorative materials via affecting organic matrix inducing surface roughness affecting light reflection [42, 64] The pH of both tea and red wine were acidic as reported in the literature: The pH of the tea was 4.9 [65], while that of red wine was 3.3 [52]. This is in contrast to the neutral pH of saliva, in addition, the increase in gloss may be due to the smooth surface obtained by the film of saliva adsorbed on the surface of the specimen [66].

There was no significant difference in the gloss of the specimens before aging (immersion and thermocycling); this may be due to the standardization in the specimens̕̕ preparation. Yet after aging, immersion generally resulted in lower gloss values compared with the thermocycling. Again, this may be attributed to the static continuous nature of immersion, versus the dynamic intermittent effect of thermocycling as discussed previously. Further investigations on the effect of aging in different beverages on the translucency and surface properties of Omnichroma single-shade restorative material are recommended.

## Conclusion

Within the limitations of the current study, it can be concluded that both resin-composites; the single-shade (Omnichroma) and multi-shade (Filtek Z350-XT), displayed unacceptable discoloration and gloss reduction after artificial-aging in tea and red-wine by immersion or thermocycling simulating one-year clinical-service. Compared to thermocycling, immersion had more pronounced aging effect when detecting color and gloss of dental restorations.

## Data Availability

All data generated or analyzed during this study are included in this published article in the form of tables and figures. The raw data that support the findings of this study are available upon request from the corresponding author.
